# Does the Use of Dihydroartemisinin-Piperaquine in Treating Patients with Uncomplicated *falciparum* Malaria Reduce the Risk for Recurrent New *falciparum* Infection More Than Artemether-Lumefantrine?

**DOI:** 10.1155/2014/263674

**Published:** 2014-06-19

**Authors:** Wisdom Akpaloo, Edward Purssell

**Affiliations:** ^1^AngloGold Ashanti Hospital, P.O. Box 10, Obuasi, Ghana; ^2^Florence Nightingale School of Nursing and Midwifery, King's College London, London SE1 8WA, UK

## Abstract

Malaria contributes significantly to the global disease burden. The World Health Organization recommended the use of artemisinin-based combination therapies (ACTs) for treatment of uncomplicated *falciparum* malaria a decade ago in response to problems of drug resistance. This review compared two of the ACTs—Dihydroartemisinin-Piperaquine (DP) and Artemether-Lumefantrine (AL) to provide evidence which one has the ability to offer superior posttreatment prophylaxis at 28 and 42 days posttreatment. Four databases (MEDLINE, EMBASE, Cochrane Database and Global Health) were searched on June 2, 2013 and a total of seven randomized controlled trials conducted in sub-Sahara Africa were included. Results involving 2, 340 participants indicates that reduction in risk for recurrent new *falciparum* infections (RNIs) was 79% at day 28 in favour of DP [RR, 0.21; 95% CI: 0.14 to 0.32, *P* < 0.001], and at day 42 was 44% favouring DP [RR, 0.56; 95% CI: 0.34 to 0.90; *P* = 0.02]. No significant difference was seen in treatment failure rates between the two drugs at days 28 and 42. It is concluded that use of DP offers superior posttreatment prophylaxis compared to AL in the study areas. Hence DP can help reduce malaria cases in such areas more than AL.

## 1. Introduction

Malaria has attracted global attention as one of the world's leading major diseases due to its high morbidity and mortality. The World Health Organization (WHO) estimated that about 3.3 billion people are at risk of malaria because they live in malaria endemic areas [1] and about 300–500 million malaria cases are reported globally each year [2]. Malaria is also responsible for almost a million deaths yearly [2, 3]. The disease is strongly associated with poverty because it disengages patients from carrying out meaningful economic activities during attacks and it also consumes huge expenditure budgets. It is estimated that as high as 40% of public health expenditure is spent on malaria alone in endemic countries [4]. Malaria is a febrile disease caused by parasites of the genus* Plasmodium *which are transmitted to susceptible persons through the bite of infected female* Anopheles* mosquitoes.

The burden of malaria is greatest in sub-Sahara Africa where approximately 80% of the global malaria cases as well as 90% of fatalities occur [1]. Children less than 5 years are the most severely affected accounting for 86% of the deaths [5]. To this extent, malaria has been recognized as an impediment to achievement of the United Nation's Millennium Development Goal (MDG) 4 which targets at the reduction of child mortality [6]. Therefore, without effective prevention and control of malaria (which is the MDG 6), it will be difficult to achieve the MDG 4.

In order to minimise the effects of malaria, prompt clinical diagnosis and effective treatment are needed [7–9] beside the preventive measures such as use of insecticide treated nets (ITN) and indoor residual spraying (IRS) with insecticides. However, prompt malaria treatment efforts are being threatened by widespread problem of antimalaria drug resistance [1, 6, 10]. For instance,* Plasmodium falciparum* had developed resistance to chloroquine which was the most affordable and readily available antimalaria drug in Africa [10]. As a result, the WHO recommended artemisinin-based combination therapy (ACT) for treatment of uncomplicated* falciparum* malaria [11, 12]. This type of treatment makes use of combination of an artemisinin derivative with a partner drug. The artemisinin derivatives are fast acting drugs capable of rapid clearing of the* falciparum* parasites during treatment [12, 13] but have short half-lives which render them almost unsuitable for use as single therapeutic drugs because of risk for drug resistance which increases as plasma levels fall [14].

Following the WHO recommendation, most sub-Sahara African countries have changed their antimalaria drug policy to the use of the ACTs [15]. Artemether-Lumefantrine (AL) is one of the ACTs commonly used as first-line drug for treatment of uncomplicated* falciparum* malaria in the sub-Sahara African countries. Another ACT—Dihydroartemisinin-Piperaquine (DP)—was also recently recommended by the WHO for use [12]. However, DP is not widely used in sub-Sahara Africa compared to the use of AL [1]. The half-lives of the partner drugs in the ACTs vary across the different ACT drugs available—see Table 1 [14]. The partner drug in AL, Lumefantrine, has a half-life of ~5 days while the partner drug in DP, Piperaquine, has a half-life of ~5 weeks, the longest of all the ACTS. As a result, it is being speculated that DP could be the ACT with greatest posttreatment prophylactic efficacy due to the longer half-life and sustained plasma levels of Piperaquine compared to other ACTs such as AL [16]. However, little is known about the relative extent to which these two ACTs exert their prophylactic effect after treatment. It is not yet clear whether the sheer longer half-life of Piperaquine in DP could translate into a better prophylaxis and to what extent compared to Lumefantrine in AL. No review had comprehensively compared these two ACTs head-to-head to determine their relative prophylactic effectiveness. Earlier reviews [17, 18] had not explored prophylaxis specifically in detail.

Olliaro and colleagues found that recurrent new infections (RNIs) were associated with higher risk for development of symptomatic disease (malaria) than recrudescence parasitaemia [19]. In contrast, other researchers had found that rather recrudescence parasitaemia carries greater risk for worse haematological outcome for patients [20]. These two studies were not conducted on ACTs but the most important issue at stake here is that both recrudescence and new infections are associated with various risks to patients; hence, prevention of their occurrence should be of much concern. This current review aimed at synthesizing available primary studies conducted in sub-Sahara Africa to determine whether use of DP reduces incidence of new* falciparum* infections (RNIs) and treatment failure more than use of AL within 28 and 42 days after treatment. A clear knowledge in this regard would offer a better guidance to country antimalaria drug policies in sub-Sahara Africa. The main review question is, “Does the use of DP in treating uncomplicated* falciparum* malaria reduce the risk for recurrent new* falciparum* infections and treatment failure more than AL?” The review tested the null hypothesis that there is no difference between the two drugs.

## 2. Materials and Methods

### 2.1. Definition of Terms

For purposes of this review, “recurrent new* falciparum* infection” (RNI) is defined as new infections which patients acquired by day 28 or 42 following treatment for uncomplicated* falciparum* malaria using DP or AL. “total treatment failure” (TTF) refers to recrudescence recorded by day 28 or day 42 (Tables 5 and 6) after treatment, where recrudescence refers to the “old” or the original parasites for which the antimalaria treatment was initiated but could not be cleared by the antimalaria drug and the disease (symptomatic malaria) has returned. The new infections must have been differentiated from the “old” infections in the primary studies by use of polymerase chain reaction (PCR). Prophylactic effectiveness used in this review refers to the ability of the ACT drug (DP or AL) to prevent the occurrence of new* falciparum* infections within 28 or 42 days after treatment with either drug (Tables 7 and 8).

### 2.2. Inclusion and Exclusion Criteria

Studies were included in the review if they were randomized controlled trial (RCT) conducted in sub-Sahara Africa and had compared the two drugs (DP and AL) head-to-head for treatment of uncomplicated* falciparum* monoinfections only. All patients recruited for the primary studies must have been confirmed through laboratory investigation to be infected with* P. falciparum* only and must not have shown signs of severe malaria or be suffering from co-febrile infection at the time of enrolment. The trial must be conducted according to the WHO protocols for antimalaria drug efficacy study. Studies which included pregnant women were excluded.

### 2.3. Database Search Strategy

An electronic search to locate relevant published studies was conducted in four main databases, namely, EMBASE, MEDLINE, Cochrane Library, and Global Health, using the search strategy presented in Table 2. The final database search for the review was conducted in the selected databases on June 2, 2013, as follows: EMBASE: from 1980 to 2013 week 22; MEDLINE: from 1946 to May 2013 week 4; Global Health: from 1973 to 2013 week 21.

**Figure 1:**
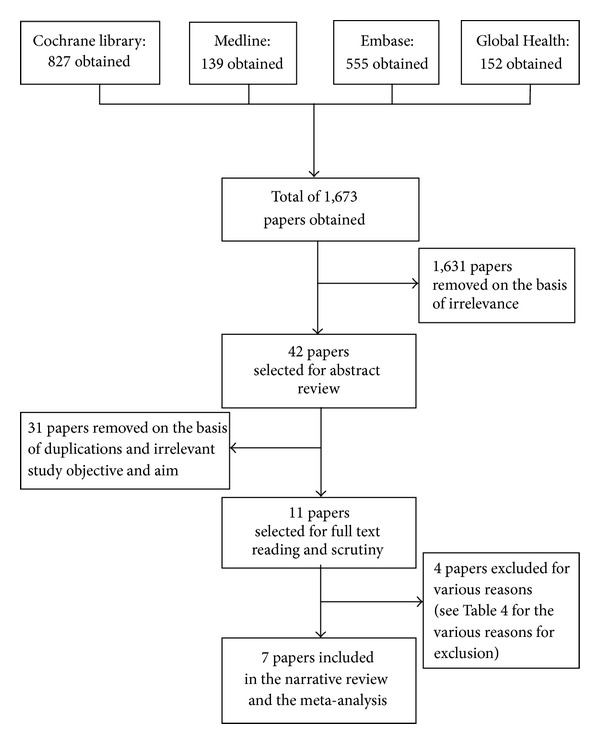
The preferred reporting items for systematic reviews and meta-analysis (PRISMA) flow diagram showing database search results and selection stages.

The main terms in the review question were structured into the PICO (population, intervention, comparison, and outcome) format and all corresponding synonyms identified. All main terms in the review question were searched under medical subject heading (MeSH terms) and also as free texts. Each MeSH term search was “exploded” and all subheadings were included. All other terms (synonyms of the main terms) were searched as free texts. Search results of terms under the same category were brought together using the Boolean operator “OR,” while the Boolean operator “AND” was used finally (once) to bring all “OR” results together. The final search result after use of the Boolean operator “AND” was limited to studies conducted on humans and have been published in English language—see Table 3. Only terms in the population, intervention, and comparison categories were used during the search because the reviewers were of the view that further inclusion of terms in the outcome category in the search might lead to exclusion of some important studies. All search results were reviewed by WA and cross-checked by EP and studies that met the inclusion criteria selected. Due to the limited time available for the review, no trial author was contacted for further clarification on the trials and, as a result, articles with instances of unclear or missing data were excluded. The preferred reporting items for systematic reviews and meta-analysis (PRISMA) flow diagram [21] has been used to summarize study selection process—see Figure 1.

### 2.4. Assessment of Quality (Risk of Bias) of Included Studies

The methodological quality of the selected studies was assessed using the “Cochrane Collaboration's tool for assessing risk of bias” [22]. The assessment tool covers six domains for assessing internal validity of studies. These domains include sequence generation, allocation concealment, blinding of participants or outcome assessors, incomplete outcome data, selective outcome reporting, and other sources of bias [22].

### 2.5. Data Extraction and Analysis

All data were extracted as binary data in the form of number of participants who experienced the event of interest and total number of participants in each study group. The outcomes of interest included recurrent new* falciparum* infections (RNIs) and total treatment failure (TTF) reported by day 28 and 42. Data on new infection and treatment failure were extracted using the differentiated PCR results recorded in the studies. Data extraction was done by author WA and figures were cross-checked by author EP for accuracy. Instances of disagreement on the accuracy of the extracted figures were resolved by extensive discussions and explanations and then agreement is finally reached on which figure is accurate.

Data analysis was done using the Comprehensive Meta-Analysis programme (CMAP) version 2.0 [23]. Effect sizes were calculated in risk ratio (RR) at 95% confidence interval (CI). Results of DP group relative to AL group were regarded as statistically significant if the 95% CI did not include number of no effect, which is 1. Absolute relative difference (ARD) and number needed to treat (NNT) were calculated [24]. The grading of recommendations assessment, development, and evaluation (GRADE) system (Table 9) was used to assess level of evidence quality [25, 26]. The meta-analyses were done in the random effects model. This model operates on the premise that each trial included in the analysis has estimated an effect size peculiar to its study population and that any difference in effect estimation across the studies is due to both random error and heterogeneity [27]. Statistical heterogeneity was assessed to determine *I*-squared (*I* ^2^), the *Q*-value, and the accompanying *P* values [27, 28]. The *I* ^2^ values were interpreted as follows: *I* ^2^ values less than 50% were regarded as low, values ≥ 50% were regarded as moderate, and an *I* ^2^ above 75% was interpreted as high or substantial heterogeneity [24, 28]. The level of statistical significance for heterogeneity *P* value was set at 10% [28].

## 3. Search Result

A total of 1,673 records were retrieved from all the database searches combined and 1,631 records (97.5%) were excluded initially because they have irrelevant study titles. Abstract of the remaining 42 publications (2.5%) were read, after which 11 (26.2%) were further selected and subjected to thorough full text reading and scrutiny. Seven studies [29–35] were finally selected for inclusion in the review.

The remaining four [15, 36–38] were excluded for various reasons (see Table 3 for reasons for exclusion).  Table 4 contains general description of selected studies.

### 3.1. Risk of Bias Assessment Results of Selected Studies

#### 3.1.1. Adequate Sequence Generation and Allocation Concealment

**Figure 2:**
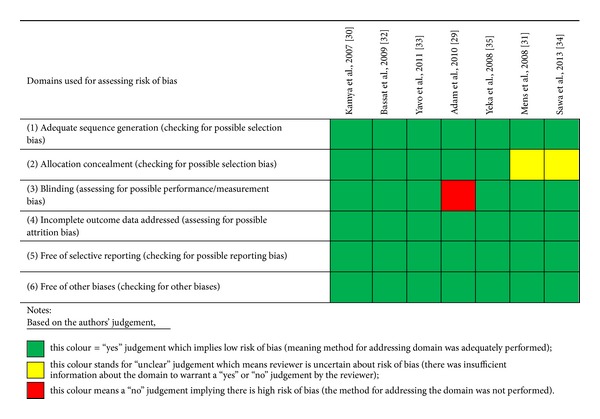
Result of risk of bias assessment for included studies.

Sequence generation was done adequately in all the studies and was judged to pose a low risk of bias. Four of the trials used computer generated random list done by an off-site investigator. One trial [29] reported use of block randomisation while the remaining two [32, 34] used stratified random lists generated by off-site investigators (Figure 2). Five trials [29, 30, 32, 33, 35] used adequate allocation concealment methods and were judged to be at low risk for selection bias because the researchers used sequentially numbered, sealed opaque envelops to obscure treatment group before allocation. The concealment process was judged to pose an unclear risk of bias in only two of the studies [31, 34] for lack of clear information.

#### 3.1.2. Blinding

Blinding was judged to be adequate (low risk of bias) in six studies [30–35] because all laboratory personnel and trial investigators involved in assessing outcome measures were blinded to the treatment allocation of the study participants. This was deemed to pose low risk of bias to parasitological outcome measures for treatment failure and rate of new infections. One study [29] did not report on blinding and was judged to pose high risk of measurement and performance bias. There was no blinding for participants and nurses in four studies and this was considered to pose rather high risk of bias to the outcome measures of adverse clinical events (side effects of the drugs) which were not the focus of this review.

#### 3.1.3. Inclusion of All Participants in the Final Analysis

All the trials selected addressed incomplete outcome data and were rated to be of low risk for attrition bias. More than 90% of randomised participants were included in final analysis in all the trials and this was deemed adequate. The highest attrition rate was 7.6%.

#### 3.1.4. Other Sources of Bias

The reviewer identified no other important source of bias.

## 4. Findings

### 4.1. Total Treatment Failure (TTF) at Day 28

Result on total treatment failure (TTF) due to recrudescence was obtained from six studies and involved a total of 3,172 patients. Of this, 1,861 participants received DP, out of which 107 (5.7%) experienced treatment failure at day 28. On the other hand, 1,311 received AL, of which 80 (6.1%) experienced treatment failure. The pooled RR yielded 0.453, 95% CI: [0.203 to 1.012, *P* = 0.05]. This implies that there was an average of 55% reduction in risk for TTF at day 28 in favour of DP treatment; largest plausible reduction possible was 80%; however, the upper boundary of the 95% CI includes the number of no effect (1) and a risk for harm of 1.2%.

**Figure 3:**
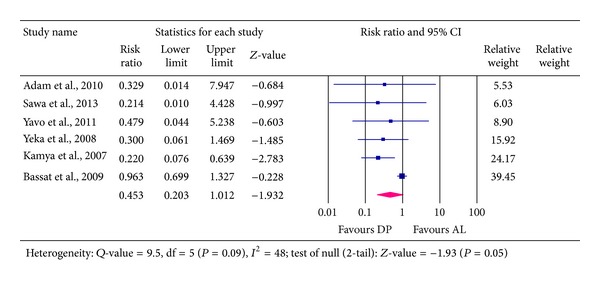
Analysis for Dihydroartemisinin-Piperaquine versus Artemether-Lumefantrine for outcome of total treatment failure (PCR- corrected) at day 28.

Extent of heterogeneity was 47% (*I* ^2^ = 47, *P* = 0.09, *Q*-value = 9.5, df = 5) and this level of heterogeneity was considered to be low [28]—see Figure 3. The pooled ARD was 0.016, 95% CI [0.030–0.002, *P* = 0.02], which means that for every 63 patients treated with DP, one case of treatment failure was prevented, which would have occurred if such patients had received AL treatment.

### 4.2. Total Treatment Failure (TTF) at Day 42

**Figure 4:**
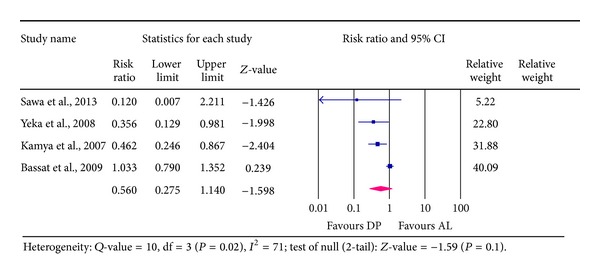
Analysis for Dihydroartemisinin-Piperaquine versus Artemether-Lumefantrine for outcome of total treatment failure (PCR-corrected) at day 42.

Data on TTF at day 42 was extracted from four studies involving 2, 662 participants, out of which treatment failure was reported in 274 participants representing 10.3%. A total of 1,598 participants were those treated with DP and 161 of them (10.1%) experienced treatment failure. Those who received AL treatment were 1,064 participants out of which 113(10.6%) experienced failure, an indication that DP was associated with a marginal reduction in treatment failure compared to AL. The pooled estimate for the RR was 0.560 with 95% CI of [0.275 to 1.140, *P* = 0.1]. This implies that there was an average reduction in risk of treatment failure of 44% in favour of DP treatment compared to AL, with the highest plausible reduction in risk of up to 73%. However, the upper boundary of the 95% CI includes number of no effect (1) and includes 14% harm of failure. Test for heterogeneity in random effect model shows high statistical heterogeneity of 71% across the studies (*Q* =10, df (*Q*) = 3, *I* ^2^ = 71%, *P* = 0.02)—see Figure 4.

### 4.3. Recurrent New Infections (RNIs) Reported at Day 28

**Figure 5:**
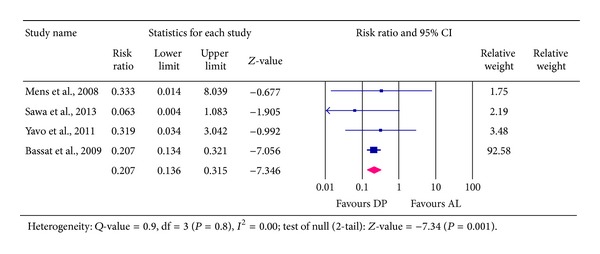
Analysis for Dihydroartemisinin-Piperaquine versus Artemether-Lumefantrine for outcome of recurrent new* falciparum* infection at day 28.

Results from four studies involving a total of 2,340 patients showed that 104 of the patients (representing 4.4%) acquired new* falciparum* infections within the 28 day period after treatment. Out of the total number, 1,433 participants received DP, of which 28 patients representing 2% had new infections. In the AL group, 907 patients received the treatment and 76 (8%) acquired new infections—an indication that rate of new* falciparum* infection was higher among those treated with AL than those treated with DP. Pooled RR was 0.207, 95% CI [0.136 to 0.315, *P* < 0.001]. This indicates that risk associated with a patient getting new infections at day 28 was significantly reduced averagely by 79% in favour of DP treatment. The lowest plausible reduction was 69% and highest was 86%; the difference was statistically significant (*P* < 0.001)—Figure 5.

### 4.4. Recurrent New Infections (RNIs) Detected at Day 42

**Figure 6:**
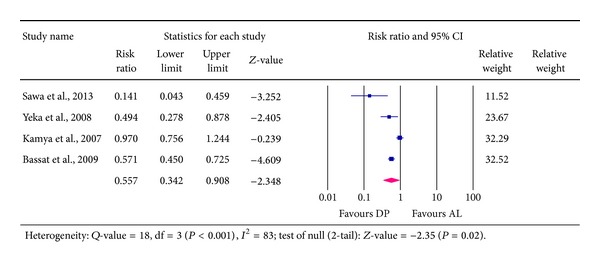
Analysis for Dihydroartemisinin-Piperaquine versus Artemether-Lumefantrine for outcome of recurrent new* falciparum* infection at day 42.

Results extracted from four studies involving a total of 2,662 patients indicated that 455 patients representing 17% experienced new infections with* falciparum* parasites. Those treated with DP were 1,598, out of which 218 (14%) acquired new* falciparum* infections. On the other hand, a total of 1,064 received AL, of which 237 (22%) experienced new infections. The pooled RR was 0.557, 95% CI [0.342 to 0.908]. This means that the risk of a patient acquiring new* falciparum* infection at 42 day following treatment was averagely reduced by 44% in favour of DP compared to AL—see Figure 6. The 95% CI was 0.342 to 0.908, suggesting that the actual effect could be anywhere between 9 and 66%; the difference was statistically significant, *P* = 0.02. There was a substantial heterogeneity (extent was 83%) associated with this result (*I* ^2^ = 83, *Q*-value = 18, df = 3, *P* < 0.001).

## 5. Discussion

This review compared two ACTs—Dihydroartemisinin-Piperaquine (DP) and Artemether-Lumefantrine (AL)—to determine which has the greatest effect in reducing recurrent new* falciparum* infections (RNIs) and treatment failure. The null hypothesis which was tested was that there is no difference between the two drugs in reducing risk of treatment failure and recurrent new* falciparum* infections at days 28 and 42. However, the overall evidence gathered suggests that patients who were treated with DP experienced less total treatment failure (TTF) and less recurrent new* falciparum* infections (RNIs) than those who received AL.

Majority of studies included in this review were conducted in East Africa and only few were carried out in West Africa and therefore findings and conclusion are more applicable to countries in the East Africa region. The evidence synthesized indicates that both DP and AL are effective in preventing treatment failure at days 28 and 42 after treatment. However, DP reduced treatment failure higher than AL at 28 and 42 days but the difference in magnitude of failure reduction between the two drugs is clinically marginal and not statistically significant. This finding concurs with that of earlier review conducted on studies done in Asia, America, and Africa [18], in which DP was found to be associated with an appreciable efficacy of cure rate compared to non-ACT and other ACT drugs. The average percentage of risk reduction in favour of DP compared to AL in this review decreased from 55% at day 28 to 44% at day 42. This confirms the assertion by Yeka and colleagues that as length of followup increases, difference between DP and AL in their ability to prevent treatment failure in high endemic areas becomes insignificant [35]. This observation is attributed to the overwhelming rate of new infections in such areas which may have outweighed the efficacy of Piperaquine coupled with the decreasing concentration of Piperaquine in the blood stream over time.

It has been speculated that DP could offer a greater posttreatment prophylaxis (PTP) than other ACTs due to its longer half-life by which it could exert longer efficacy against newly infecting parasites and reduce risk for development of both clinical malaria and resistance parasite strains [14]. However, the extent to which DP could offer prophylaxis (PTP) by preventing clinical malaria due to new infections has not been clearly specified compared to other ACTs such as AL.

Result on prevention of recurrent new* falciparum* infections (RNIs) in this review demonstrates that DP offered greater and significant PTP for patients against RNI than AL. Average percentage reduction in risk for RNI was up to 79% at day 28 in favour of DP and up to 44% at day 42 in favour of DP. There is, however, substantially significant statistical heterogeneity (extent was 83%) associated with pooled result on RNI at day 42 in this review. The possible source of this heterogeneity is attributed to the variability in malaria infection transmission rate of the various study sites; some of the studies were conducted in settings where rate of malaria transmission intensity was very high while other studies were conducted in areas of relatively low malaria transmission intensity. Thus, study settings with very high transmission intensity are likely to be associated with higher rates of* Anopheles* mosquito bites which will result in recurrent new infections more than settings with low transmission intensities. Even though participants were provided with insecticide treated nets, it cannot be ascertained whether patients actually slept in the nets to prevent mosquito bites after treatment and this could also affect the proportion of patient who experienced recurrent new infections.

The superiority of DP in offering this higher level of posttreatment prophylaxis (PTP) is attributable to the longer half-life of Piperaquine (which is ~5 weeks) compared to the shorter half-life (of about 4 to 5 days) of Lumefantrine in AL [14]. The higher PTP of DP will be of significant importance for areas of higher malaria transmission intensities more than for areas of low transmission intensities which are associated with lower frequency of acquiring malaria due to new infections. However, in spite of the benefit of higher PTP of DP it must also be pointed out that the longer half-life of Piperaquine is likely to pose high risk for faster development of drug resistant strains of the* falciparum* parasites to DP [14]. This is thought to be possible because the new infecting parasites will be exposed to subtherapeutic (low) concentrations of Piperaquine over time. This low concentration will not be able to eliminate the parasites completely and will provide an opportunity for development of resistant strains. Therefore, any attempt to promote more utilisation of DP for the benefit associated with its higher PTP must be weighed against the possible risk for development of resistant strains because of potential public health dangers associated with drug resistant parasites. Notwithstanding, the risk for development of drug resistant parasites could be minimized if more than one ACT drug is used as a first-line drug for treatment of uncomplicated malaria in a particular area [15].

The use of more than one drug as a first-line ACT may prevent the possibility of the parasites becoming adjusted to one overused-first-line ACT. There is, therefore, the need for encouraging the use of multiple first-line drugs and constant monitoring of efficacy of the ACT drugs to determine development of resistant strains at early stages.

### 5.1. Clinical and Policy Implications of Findings

Using the GRADE criteria for rating evidence quality [26], the quality of evidence obtained regarding superiority of DP over AL in preventing TTF at day 28 was rated moderate quality while that of day 42 was rated low quality. Evidence obtained on prophylactic superiority of DP over AL in preventing RNI at day 28 was rated high quality while that of day 42 was rated moderate quality. High quality GRADE evidence rating means that the authors are very confident that the true effect lies close to the average estimated while moderate quality rating means that authors' confidence in the estimate is moderate which means that the true effect size is likely to be close to the average estimate, but there is also likelihood that there could be substantial difference [26].

It is worth indicating that treatment failure (TTF) and recurrent new infections (RNIs) are of great importance and concern to patients due to the negative socioeconomic impact of clinical malaria on patients. Malaria imposes high economic burden on patients by preventing them from working when the disease attacks them and this slows work force productivity. Malaria is highly prevalent in sub-Sahara Africa and it is undeniable that patients acquiring frequent recurrent new infections contribute to the high number of cases. Therefore, preventing RNIs means that an appreciable proportion of the population would remain healthier for productive economic activities and this could help reduce the malaria expenditure burden on the healthcare system and on the continent as well as on donor agencies. Considering these reasons above and level of relative reduction in risk for TTF and RNI in favour of DP together with the quality of the evidence obtained, these findings are considered to be of clinical and country malaria policy significance.

### 5.2. Strengths of the Review

All studies included in the review were RCTs which is appropriate for answering clinical intervention questions. The methodological quality of the selected studies was generally high and had been rated to pose low risk of bias. Meta-analysis was done in random effects model to integrate extracted data for better interpretation and all these are considered as strengths of the review. Data extracted from the various studies were done by AW and cross-checked by EP to ensure accuracy and prevent individual bias.

### 5.3. Limitations of the Review

There was no attempt to locate studies in the grey literature and other sources apart from those indexed in the four databases searched. Search results were limited to studies published in English language alone and therefore excluded other equally valuable articles which might have been published in other languages such as French. All of these carry a potential risk of selection bias which could undermine the completeness of the review data and weaken findings and conclusion. There are variations associated with the studies. Firstly the studies were conducted in different sites that have different malaria transmission intensities. Studies also recruited participants with different age groups. Also, majority of the included studies were conducted in countries in East Africa which weakens generalisation of findings to other parts of sub-Sahara Africa; hence this review result and conclusion are most valid for countries in East Africa. There is high statistical heterogeneity associated with some estimates; hence each pooled effect estimate must be seen as an average representing different estimates which are peculiar to each study population, bearing in mind, however, that effect directions were similar and, in most cases, favoured DP (see forest plots in Figures 3–6).

## 6. Conclusion

This systematic review compared Dihydroartemisinin-Piperaquine (DP) and Artemether-Lumefantrine (AL) and aimed at identifying which one has greater ability to reduce total treatment failure (TTF) and incidence of recurrent new* falciparum* infections (RNIs) in high transmission areas in sub-Sahara Africa. The results showed that participants treated with DP compared to AL experienced lesser risk for TTF at days 28 and 42.

On the other hand, DP offers a significant posttreatment prophylaxis against recurrent new* falciparum* infections superior to that of AL. The average percentage reduction in risk for the incidence of RNI was up to 79% at day 28 in favour of DP [RR, 0.21; 95% CI: 0.14 to 0.32, *P* < 0.001] and 44% in favour of DP at day 42 [RR, 0.56; 95% CI: 0.34 to 0.90; *P* = 0.02]. It is, therefore, concluded that treatment of uncomplicated* falciparum* malaria using Dihydroartemisinin-Piperaquine (DP) in high transmission areas in sub-Sahara Africa (especially East Africa) could result in an average reduction in risk for recurrent new* falciparum* infections of up to 79% and 44% within 28 and 42 days, respectively, compared to Artemether-Lumefantrine (AL). And this implies that use of DP can help reduce burden of malaria in such areas more than AL. However, both DP and AL have similar effectiveness in preventing treatment failure though DP has marginal benefit over AL.

## 7. Recommendation

It is recommended that the antimalaria drug policy in countries especially in East Africa should be streamlined to include use of Dihydroartemisinin-Piperaquine alongside Artemether-Lumefantrine and other ACTs in countries where it is found to be effective but this must be done bearing in mind the potential risk for development of resistant* falciparum* strains. More studies should be conducted in other parts of the sub-Sahara Africa such as West Africa to determine stronger evidence which will be more applicable to countries in that area.

It has been identified that the current study protocol by the WHO regarding antimalaria drug efficacy research does not incorporate specific outcome measure on recurrent new infections. It is, therefore, recommended that measurement of rate of recurrent new infections should be incorporated into future guidelines, and future trial investigators should make direct assessment on it.

## Figures and Tables

**Table 1:** Plasma half-lives (*T*_1/2_) of drugs used in some common ACTs.

Antimalarial drug	*T*_1/2_^1^ of artemisinin derivative	*T*_1/2_ of partner drug
Artemether-Lumefantrine (AL)	~3 hrs	4-5 days
Artesunate-Amodiaquine (AMQ)	<1 hr	9–18 days
Dihydroartemisinin-Piperaquine (DP)	45 min	~5 weeks Source: [14]

^1^ *T*_1/2_ (half-life): it is the length of time required for half of the concentration of the ACT drugs in the body of a patient to be eliminated.

**Table 2:** The electronic search strategy.

#1. Malaria [MeSH]-search “exploded” and all subheadings included	
#2. Malaria [free text]	
#3. Uncomplicated malaria [free text]	
#4. Uncomplicated falciparum malaria [free text]	
#5. Simple malaria [free text]	
#6. #1 OR #2 OR #3 OR #4 OR #5	
#7. Dihydroartemisinin plus Piperaquine [MeSH]-search “exploded” and all subheadings included	
#8. Dihydroartemisinin plus Piperaquine [free text]	
#9. Dihydroartemisinin-Piperaquine [free text]	
#10. Dihydroartemisinin [free text]	
#11. Arteether [MeSH]-search “exploded” and all subheadings included	
#12. Dihydroartemisinin [MeSH]-search “exploded” and all subheadings included	
#13. #7 OR #8 OR #9 OR #10 OR #11 OR #12	
#14. Artemether plus Lumefantrine [MeSH]-search “exploded” and all subheadings included	
#15. Artemether plus Lumefantrine [free text]	
#16. Artemether-Lumefantrine [free text]	
#17. Artemether Lumefantrine [free text]	
#18. Arteether [MeSH]-search “exploded” and all subheadings included	
#19. Coartem [free text]	
#20. Riamet [free text]	
#21. Coarteme [free text]	
#22. Co-artemether [free text]	
#23. #14 OR #15 OR #16 OR #17 OR #18 OR #19 OR #20 OR # 21 OR 22	
#24. #6 AND #13 AND #23	
#25. Limit #24 to human and English language	

**Table 3:** Table of excluded studies and reasons for exclusion.

Study name	Design, country	Sample size	Reasons for exclusion
Nambozi et al., 2011 [36]	RCT, Zambia	304	The study has been reported as part of the larger multicentric study of Bassat et al., 2009, already included in the review

Arinaitwe et al., 2009 [15]	RCT, Uganda	351	Some of the study participants were infected with other species of Plasmodium. The reviewer was only interested in primary studies in which only *P. falciparum* infections were treated.

Verret et al., 2011 [37]	RCT, Uganda	292	This study is a subanalysis of the main study of Arinaitwe et al., 2009, which was also excluded from the review with reasons as the above.

Katrak et al., 2009 [38]	RCT, Uganda	246	There was no data on treatment failure; only adverse events were presented. The main aim of the study was to investigate safety of study drugs among HIV-infected and HIV-uninfected children, which is not relevant for this review.

**Table 4:** Description of major characteristics of studies included.

Reference	Methodology	Participants	Intervention	Outcomes measured	Researchers' finding and conclusion	Reviewer's comments
Adam et al., 2010 [29]	*Study design*: RCT *Country*: Sudan *Setting*: Elmouraf Health Centre in Sinnar *Malaria transmission rate of area*: moderate *Participant allocation*: it was by block randomization and concealment of allocation was by use of sealed opaque envelopes containing treatment	*Mean age*: DP group: 9.8 years; AL group: 8.4 years. *Sample size at start of study*: 160 patients (80 in each group) *Final analysis*: it was based on 149 patients (75 in DP and 74 in AL); attrition rate was 6.9% *Inclusion criteria*: patients aged ≥6 months; axillary temperature ≥37.5°C; monoinfection with *P. falciparum*; informed consent and ability of patient to take oral medications *Exclusion criteria:* signs of severe malaria; presence of other febrile illness; patients who were allergic to any of study drugs; pregnant women and patients who had used the study drug in preceding 28 days	*Experimental group:* oral treatment with DP (Duocotexin)/40 mg D/320 mg P tablet for adults and 20 mg D/160 mg P for children were given according to weight as follows: 5–9 kg: half tablet; 10–19.9 kg: one tablet; 20–40 kg: two tablets; or >40 kg: three “adult” tablets or two“child” tablets (once daily for 3 days) *Control group*: oral AL (Coartem) tablets 20 mg A/120 mg L were used according to weight as follows: 5–14 kg: one tablet; 15–24 kg: two tablets; 25–34 kg: three tablets; and >35 kg: four tablets (twice daily for 3 days)	*Primary outcome:* adequate clinical and parasitological response (ACPR) by day 28 *Secondary outcome:* frequencies of early treatment failure (ETF) by day 3 of treatment; late clinical response (LCR) by day 28; late parasitological response (LPR) by day 28; gametocyte carriage; adverse events recorded during treatment	The cure rate of participants by day 28 was found to be 100% for DP and 98.7% for AL This implies that both DP and AL were effective and safe for treating uncomplicated *P. falciparum* malaria but the DP might be preferred to AL due to its simple once daily dosing regimen compared to twice daily dose required for AL	**Strengths:** ethical clearance was acquired; written consent was obtained from guardians; random allocation process was used to allocate participants into study groups with concealment of allocation process to prevent selection bias; the two study groups were reported to be similar before start of study; both study groups were treated equally and differ only in study drugs served; all treatments were served at the health centre to ensure compliance; attrition rate was 6.9% which is deemed to be insignificant to affect internal validity of study results. Length of followup was 28 days which is in consonance with the WHO standards for efficacy studies on antimalaria drugs. All outcome measures were also according to the WHO protocol. **Weaknesses:** (1) there was no information in the paper indicating that power calculation was done which would ascertain sample size adequacy. This means that there is the likelihood that type II error could have been committed, that is, the inability to detect differences in the efficacy of the two drugs even if it existed. (2) No information was given in the paper on blinding of outcome assessors; hence there is likelihood of measurement bias in this study.
Kamya et al., 2007 [30]	*Study design:* RCT *Country:* Uganda *Malaria transmission status of area*: very high intensity *Participant allocation*: it was by random allocation using random list generated by off-site investigator using computer and concealment of allocation was ensured using sealed opaque envelopes containing treatment groups	*Mean age:* 1.8 years for DP group and 1.5 years for AL group *Sample size at start of study*: 421 (211 in DP group and 210 in AL group) *Number included in final analysis*: 417 (99%) (209 in DP and 208 in AL) *Inclusion criteria*: children between 6 months and 10 years old; weight ≥5 kg; history of fever in last 24 hrs or axillary temperature ≥37.5°C; no allergy to study medications; no concomitant febrile illness; informed consent; no sign of severe malaria and infection with *P. falciparum* only	*Experimental group*: DP (Duocotexin 40 mg D/320 mg P) tablets were administered as 6.4 and 51.2 mg/kg of D and P, respectively, given in daily doses for 3 days. *Control group:* AL (Coartem 20 mg, Artemether/120 mg, and Lumefantrine) tablets were administered according to weight as follows: one (5–14 kg), two (15–24 kg), three (25–34 kg), or four tablets (≥35 kg). Served twice daily for 3 days	*Primary outcome* Early treatment failure (ETF) by day 3 Late clinical failure (LCF) by day 28 and 42 Late parasitological failure (LPF) Adequate clinical and parasitological response by day 42 *Secondary outcomes* Serious adverse events Common adverse events Fever clearance by day 3	Risk of recurrent *falciparum* infection was significantly lower for patients treated with DP compared to AL by day 28 [11% versus 29%; RD = 18%; 95% CI (11%–26%)] and by day 42 [43% versus 53%; RD = 9.6%; 95% CI: 0%–19%]. The conclusion was that DP was superior to AL in reducing risk for recurrent *falciparum* infection	**Strengths:** ethical clearance was acquired; written consent was obtained from guardians; participants were allocated into study groups by random allocation process and allocation process was also concealed to prevent selection bias by use of sealed opaque envelopes; power calculation was done and hence adequacy of sample was ascertained to prevent type II error; no significant difference between the study groups before start of study; both study groups were treated equally and differ only in study drugs served; attrition rate was 0.95% which is insignificant to affect study result validity. Length of followup was 42 days and was within the WHO standards. All outcome measures were according to the WHO protocol and outcome assessors were kept blind to the treatment groups of the participants. **Weaknesses:** care givers (nurses) were not blinded to treatments of participants which could be a source of performance bias
Mens et al., 2008 [31]	*Design*: RCT *Country*: Kenya *Malaria transmission status of area*: high *Participant allocation*: it was by random allocation using computer generated random list with concealment of allocation process ensured with sealed opaque envelops which contained treatment group	*Mean age*: DP group: 60 months; AL group: 52 months *Sample size at start of study:* 146 (73 for AL and 73 for DP) *Sample included in final analysis*: 134 (92%)-67 for AL; 67 for DP *Inclusion criteria*: children between 6 months and 12 years; uncomplicated *P*. *falciparum* infection; axillary temperature ≥37.5°C or history of fever. *Exclusion criteria*: infection with other *Plasmodium* spp.; severe malaria; presence of other underlying infectious conditions	*Experimental group*: oral DP tablet of 20 mg D/160 mg P (paediatric formulation) was used and was given according to weight as follows: between 4–7 kg: half a tablet; 7–13 kg: one tablet; 13–24 kg: 2 tablets; 24–35 kg: four tablets. Tablets were given daily for 3 days *Control group*: oral AL tablet, 20 mg Artemether/120 Lumefantrine was served according to bodyweight: 5–14 kg: one tablet; 15–24: two tablets; and 25–34 kg: three tablets; drug was served twice daily for 3 days	Early treatment failure at day 3 Fever clearance Adverse events Gametocyte carriage Haemoglobin level	Rate of parasite clearance for the first 3 days was slower in patients treated with DP than those who received AL, but after day 28, there was no difference between the two drugs	**Strengths:** ethical clearance was obtained; written consent was obtained from guardians; participants were allocated into study groups by process of random allocation; there was no significant difference between the study groups before start of study; both study groups were treated equally and differ only in study drugs served; attrition rate was very low (7.6%) which is deemed insignificant to affect study results. Length of followup was 28 days and was within the WHO standards. Outcome assessors were kept blind to the treatment groups of the participants **Weaknesses:** (1) no description of concealment of allocation process was given in the paper implying there could be selection bias; power calculation was not mentioned and hence adequacy of sample could not be ascertained hence there is likelihood of type II error committed in the study, that is, inability to detect differences between study treatments
Bassat et al., 2009 [32]	*Design*: Noninferiority trial *Countries:* Burkina Faso, Kenya Mozambique, Uganda, and Zambia *Malaria transmission status of areas*: high for all sites *Participant allocation*: it was done by random allocation process using computer generated stratified random list and allocation concealment ensured using sealed opaque envelops	*Sample size at start of study*: 1,548 (1038 in DP group and 510 in AL group) *Final analysis*: it was based on 1468 (94.8%) (986 for DP group and 482 for AL group) for day 28 as against 1451 (93.7%) (977 versus 474 for the groups, resp.) for day 42. *Inclusion criteria*: children between 6 and 59 months; weight >5 kg; microscopically confirmed *P*. *falciparum* monoinfection; history of fever in the preceding 24 hours or axillary temperature ≥37.5°C; informed consent *Exclusion criteria*: severe malaria; acute malnutrition; any contraindication to receive study drugs; current treatment with any antimalaria drug; presence of any concomitant infectious disease	All participants received directly observed treatment for 3 days *DP group*: two different formulations of Eurartesim tablet (20 mg D + 160 mg P or 40 mg D + 320 mg P) were used and administered according to standard bodyweight dosage of 2.25 mg/kg and 18 mg/kg of D and P, respectively *AL group*: Coartem (AL) tablet (20 mg Artemether + 120 mg Lumefantrine) was given according to bodyweight twice daily for 3 days as follows: 5–14 kg: one tablet per dose, 15–24 kg: two tablets per dose, 25–34 kg: three tablets per dose	*Primary outcome* Adequate clinical and parasitological response (ACPR) by days 28 and 42 Early treatment failure (ETF) by day 3 Late treatment failures (LTF) by days 28 and 42 *Secondary outcomes* Fever clearance rate Haemoglobin level Adverse events	The PRC-corrected cure rate by day 28 was 90.4% for patients who received DP and 90.0% for those treated with AL (*P* = 0.820), meaning the difference was not statistically significant. They concluded that both drugs have comparable efficacies in treating uncomplicated malaria in children from different settings in Africa	**Strengths:** ethical clearance was acquired; written consent was obtained from guardians; participants were allocated into study groups by process of random allocation; there was no significant difference between the study groups before start of study; both study groups were treated equally and differ only in study drugs served; attrition rate was very low (7.6%) which is insignificant to affect study results. Length of followup was 42 days and was within the WHO standards. Outcome assessors were kept blind to the treatment groups of the participants to avoid measurement bias **Weaknesses:** (1) the different settings involved might have introduced some level of difference (heterogeneity) in the result and affect their combination
Yavo et al., 2011 [33]	*Study design:* RCT *Country*: Cameroon, Cote d'Ivoire, and Senegal (multicentric) *Malaria transmission status of sites*: high *Participant allocation*: it was by random allocation, using computer generated random list with concealment ensured by use of sealed opaque envelopes containing treatment group controlled by an independent nurse	*Mean age*: experimental group: 15.6 years; control group: 13.5 years *Sample size at start of study*: 384 (187 in AL arm and 197 in DP arm) *Final analysis*: it was based on 374 patients (183 in AL and 191 in DP) *Inclusion criteria*: patient with *P. falciparum* monoinfection; fever with axillary temperature ≥37.5; patient must be ≥2 years old; informed consent; no history of allergy to study drugs; no concomitant febrile illness; no severe malaria; no treatment with an antimalaria drug in previous 3–7 days; not pregnant or nursing; and no ongoing antimalaria treatment	*Control group*: Coartem tablets (20 mg Artemether + 120 mg Lumefantrine) were administered twice daily for 3 days according to weight as follows: 5–14 kg: one tablet per dose; 15–24 kg: two tablets per dose; 25–34 kg: three tablets per dose; and ≥35 kg: four tablets per dose *Experimental group*: Duocotexin tablets (40 mg D + 320 mg P) were administered daily for 3 days per bodyweight: 5–9 kg: half tablet per dose; 10–14 kg: 3/4 tablet per dose; 15–19 kg: 1 tablet per dose; 20–24 kg: 1 + 1/4 tablet per dose; 25–29 kg: 1 + 1/2 tablet per dose; 30–34 kg: 1 + 3/4 tablet per dose; 35–39 kg: 2 tablets per dose; 40–44 kg: 2 + 1/4 tablet per dose; 45–49 kg: 2 + 1/2 tablet per dose; and >50 kg: 3 tablets per dose	*Primary outcome* Recovery rate by day 28 Early clinical failure by day 3 Late clinical failure (LCF) *Secondary outcome* Rate of fever clearance; rate of parasite clearance, and adverse clinical and laboratory events	Adjusted recovery rate was 99.5% for patients treated with DP and 98.9% for those who received AL but the difference was not statistically significant (*P* = 0.538). No early treatment failures were recorded. The investigators concluded that DP was as efficacious as AL in treatment of uncomplicated *falciparum* malaria and added that DP may be preferred by patients because of its simple daily dose regimen	**Strengths:** ethical clearance was acquired; written consent was obtained from guardians; participants were allocated into study groups by process of random allocation; all the study groups were comparable before start of treatment; both groups were treated equally and differ only in study drugs served; attrition rate was very low (2.6%) which is deemed insignificant to affect study results. Length of followup was 28 days in line with the WHO standards. Outcome assessors were kept blind to the treatment groups of the participants to present objective measurement of outcomes **Weaknesses:** (1) the different treatment sites and country could introduce variation in participants and affect homogeneity
Sawa et al., 2013 [34]	*Study design*: RCT *Country*: Kenya *Malaria transmission status of area*: moderate intensity *Patient allocation*: it was by random allocation using computer generated random list and concealment was ensured using opaque sealed envelopes containing treatment groups controlled by an independent nurse.	*Median age in years*: experimental group: 5 (3–8); control group: 5 (3–7) *Sample size at start of study:* 298 (experimental. group: 145; control group: 153) *Sample size in analysis*: 284 by day 28 (137 for exp. and 147 for control); 279 by day 42 (134 in exp. and 145 in control). *Inclusion criteria*: children between 6 months and 10 years old; history of fever in last 24 hours or tympanic temperature of ≥37.5°C; confirmed infection with *P*. *falciparum* parasite; informed consent *Exclusion criteria*: patient haemoglobin level <5 g/dL; presence of other febrile illness; infection with other *Plasmodium* species; history of adverse reaction to study drugs; signs of severe malaria	*Experimental group:* DP (Duocotexin) tablets (40 mg D + 320 mg P) were administered at a target total dose of 6.4 mg and 51.2 mg of D and P, respectively, in 3 equally divided daily doses for 3 days *Control group*: AL (Coartem) tablets (20 mg Artemether + 120 mg Lumefantrine) were administered as half a tablet per 5 kg bodyweight twice daily for 3 days.	*Primary outcome:* adequate clinical response by day 28 and 42 Early treatment failure (ETF) by day 3 Late treatment failure (LTF) by day 28 and 42 *Secondary outcomes* Gametocyte prevalence at days 28 and 42 Parasite clearance by day 3	Adequate clinical recovery by day 28 was 100% in patients treated with DP compared to 93.2% in those treated with AL with difference being statistically significant (*P* = 0.002). The cumulative risk for recurrent infection on day 42 was 3.7%; 95% CI (1.2–8.5) for DP and 20.7%; 95% CI (14.4–28.2); the difference was statistically significant. Their conclusion was that treatment with DP was associated with longer prophylactic efficacy than AL	**Strengths:** ethical clearance was acquired; written consent was obtained from guardians; the study with robust design generally. Efforts were made to reduce systematic bias: participants were allocated into study groups by process of random allocation; all the study groups were comparable with no significant differences before start of treatment; both groups were treated equally and differ only in study drugs served; attrition rate was very low and insignificant to affect study results. Length of followup was 28 days, in line with the WHO standards. Outcome assessors were kept blind to the treatment groups of the participants to ensure objective measurement of outcomes. **Weaknesses:** (1) no description or mention was made of concealment of allocation process and therefore could not be ascertained. There is, therefore, a high possibility of selection bias if concealment was not ensured
Yeka et al., 2008 [35]	*Study design*: RCT *Country*: Uganda *Setting:* Kihihi Health Centre in Kanungu *Malaria transmission status of area*: high *Patient allocation*: it was by random allocation using computer generated random list done by an off-site investigator Concealment was done by use of sealed opaque envelopes containing treatment groups	*Mean age:* experimental group: 2 years; control group: 2 years *Sample size at start of trial*: 414 (experimental group: 215 and control group: 199) *Sample during analysis*: 408 (3 participants lost in each study group) *Inclusion criteria*: children between 6 months and 10 years, monoinfection of *P*. *falciparum* parasite, no presence of other febrile illness, informed consent *Exclusion criteria*: haemoglobin level <5 g/dL, presence of signs of severe malaria	*Experimental group*: Received DP (Duocotexin) tablets (40 mg D + 320 mg P) for a target total dose of 6.4 and 51.2 mg of D and P, respectively, in 3 equally divided daily doses for 3 days. DP patients were given placebo in the evening to mimic the twice dosing for AL group *Control group*: Received AL (Coartem) tablets (20 mg Artemether + 120 mg Lumefantrine) twice daily for 3 days according to body weight as follows: 5–14 kg: one tablet per dose; 15–24 kg: two tablets per dose; 25–34 kg: three tablets per dose; and ≥35 kg: four tablets per dose All medications were served with water and patients were given milk afterwards to improve absorption	*Primary outcome* Early treatment failure by day 3 Late clinical failure at days 28 and 42 Late parasitological failure by days 28 and 42 *Secondary outcome* Rate of fever clearance Rate of parasite clearance Adverse event Presence of gametocyte	Risk for recurrent infection in patients treated with DP was significantly lower than those treated with AL (12.2% versus 33.2%), respectively. Risk difference (RD) = 20.9%; 95% CI (13.0–28.8%); *P* < 0.0001. The researchers concluded that treatment of uncomplicated malaria with DP was highly efficacious and could also be preferred more to AL because of its simple daily dosing regimen	**Strengths:** all ethical requirements were satisfied; efforts were made to reduce systematic bias as follows: participants were allocated into study groups by process of random allocation; all the study groups were similar in terms participant characteristics before start of treatment; both groups were treated equally and differ only in study drugs served; attrition rate was very low and insignificant to affect study results. Length of followup was 42 days in line with the WHO standards. All outcome assessors were kept blind to the treatment groups of the participants to present objective measurement of outcomes **Weaknesses:** (1) nurses who provided care and administered study drugs were not blinded which may be a likely source of performance bias

**Table 5:** Findings on total treatment failure (TTF), PCR-corrected by day 28.

Study name	Study groups with number of failures	Risk (*R*)	Risk ratio (RR) (95% CI)	*P* value	Interpretation for RR values
Bassat et al., 2009 [32]	DP (*N*/*N*): 100/1038 AL (*n*/*N*): 51/510	0.096 0.010	0.963 (0.699–1.327)	0.819	This means there is 3.7% reduction in risk of failure in favour of DP treatment but the result is not statistically significant. The highest possible reduction in risk was 30.1%, favouring DP.

Yavo et al., 2011 [33]	DP (*n*/*N*): 1/191 AL (*n*/*N*): 2/183	0.005 0.011	0.479 (0.044–5.238)	0.546	Treatment with DP had contributed to a point estimate of 52.1% reduction in treatment failure, with highest possible reduction of 95.6% but the result is not statistically significant.

Kamya et al., 2007 [30]	DP (*n*/*N*): 4/211 AL (*n*/*N*): 18/210	0.019 0.087	0.220 (0.076–0.639)	0.005	This result shows that DP treatment was associated with a point estimate reduction of 78% in treatment failure, with lowest and highest of the estimates being 36.1% and 92.4%, respectively, and is statistically significant.

Sawa et al., 2013 [34]	DP (*n*/*N*): 0/137 AL (*n*/*N*): 2/147	0.000 0.014	0.214 (0.010–4.428)	0.319	DP treatment had contributed to 78.6% reduction in treatment failure but it is not statistically significant. The largest plausible reduction would be 99%.

Adam et al., 2010 [29]	DP (*n*/*N*): 0/75 AL (*n*/*N*): 1/74	0.000 0.014	0.329 (0.014–7.947)	0.494	Result shows a statistically insignificant difference between DP and AL treatment in preventing treatment failure but indicates that there was a 67.1% risk of failure reduction in favour of DP treatment (1 > RR, 1 − RR = 1 − 0.329 = 0.671 × 100 = 67.1%).

Yeka et al., 2008 [35]	DP (*n*/*N*): 2/211 AL (*n*/*N*): 6/190	0.009 0.032	0.300 (0.061–1.469)	0.138	Result is not significant statistically but there was a reduction in failure in favour of DP treatment of 70%. The highest reduction possible was 93.9% in favour of DP treatment of *falciparum* malaria.

**Table 6:** Findings on total treatment failure (TTF), PCR-corrected by day 42.

Study name	Study groups with number of failures	Risk (*R*)	Risk ratio (RR) (95% CI)	*P* value	Interpretation of RR values
Bassat et al., 2009 [32]	DP (*N*/*N*): 143/1038 AL (*n*/*N*): 68/510	0.138 0.133	1.033 (0.790–1.352)	0.811	There was a marginal increase in treatment failure of 3.3% associated with DP treatment but it is not statistically significant.

Kamya et al., 2007 [30]	DP (*n*/*N*): 13/211 AL (*n*/*N*): 28/210	0.062 0.133	0.452 (0.245–0.867)	0.016	This result shows that DP treatment was associated with a point estimate reduction of 54.8% in treatment failure; with lowest and highest of the estimates being 13.3% and 75.5%, respectively, and it is statistically significant.

Sawa et al., 2013 [34]	DP (*n*/*N*): 0/134 AL (*n*/*N*): 4/145	0.000 0.028	0.120 (0.007–2.211)	0.154	DP treatment had contributed to 88% reduction in treatment failure but it is not statistically significant. The largest plausible reduction was 99.3%.

Yeka et al., 2008 [35]	DP (*n*/*N*): 5/215 AL (*n*/*N*): 13/199	0.023 0.065	0.356 (0.129–0.981)	0.045	This result is statistically significant. There was a reduction in failure in favour of DP treatment of 64.4%. The highest possible reduction was 87% and the lowest was 2% in favour of DP treatment of falciparum malaria.

**Table 7:** Findings on new *falciparum* infections detected by day 28.

Study name	Study groups with number of new infections	Risk (*R*)	Risk ratio (RR) (95% CI)	*P* value	Interpretation for the RR values
Sawa et al., 2013 [34]	DP (*n*/*N*): 0/137 AL (*n*/*N*): 8/147	0.00 0.054	0.063 (0.004–1.083)	0.057	There has been 37% reduction in new infections associated with DP treatment but reduction is not statistically significant.

Yavo et al., 2011 [33]	DP (*n*/*N*): 1/191 AL (*n*/*N*): 3/183	0.005 0.016	0.319 (0.034–3.042)	0.321	There was a 68% reduction in new infections in favour of DP treatment but result not statistically significant.

Bassat et al., 2009 [32]	DP (*n*/*N*): 27/1038 AL (*n*/*N*): 64/510	0.026 0.125	0.207 (0.134–0.321)	<0.001	This result shows 79% reduction in incidence of new *falciparum* infections in favour of DP treatment and the reduction is statistically significant. The lowest plausible reduction is 67.9% while the highest is 86.6% as defined by the 95% CI of the RR.

Mens et al., 2008 [31]	DP (*n*/*N*): 0/67 AL (*n*/*N*): 1/67	0.000 0.015	0.333 (0.014–8.039)	0.499	DP treatment was associated with a 66.7% reduction in risk for a patient to acquire new infections compared to AL. The reduction is not statistically significant.

**Table 8:** Total new *falciparum* infections detected by day 42.

Study name	Study groups with number of new infections	Risk (*R*)	Risk ratio (RR) (95% CI)	*P* value	Interpretation for the RR values
Sawa et al., 2013 [34]	DP (*n*/*N*): 3/134 AL (*n*/*N*): 23/145	0.022 0.159	0.141 (0.043–0.459)	0.001	There has been 85.9% reduction in new infection associated with DP treatment. Highest plausible reduction is 95% while lowest is 54% defined by the 95% CI. The reduction is statistically significant.

Yeka et al., 2008 [35]	DP (*n*/*N*): 16/215 AL (*n*/*N*): 30/199	0.074 0.151	0.494 (0.278–0.878)	0.016	There was 50.6% reduction in risk to acquire new *falciparum* infection in favour of DP treatment and the reduction is statistically significant.

Bassat et al., 2009 [32]	DP (*n*/*N*): 122/1038 AL (*n*/*N*): 105/510	0.118 0.206	0.571 (0.450–0.725)	<0.001	This result shows 42.9% reduction in risk of getting new infections with *falciparum* and the reduction was in favour of DP and is statistically significant. The lowest plausible reduction is 27.5% while the highest is 55% as defined by the 95% CI of the RR.

Kamya et al., 2007 [30]	DP (*n*/*N*): 77/211 AL (*n*/*N*): 79/210	0.365 0.376	0.970 (0.756–1.244)	0.811	DP treatment was associated with only 3% risk reduction for new infection and the reduction is statistically insignificant.

**Table 9:** GRADE profile of evidence quality on outcome measures.

Study characteristics			Quality assessment				Finding summary	Grading
Study design	No. of participants	Outcome measure	Limitation	Consistency	Directedness	Precision	Outcome: pooled RR (95% CI) and *P* value	Quality remark
RCT	3,172 (number of studies = 6)	Total treatment failure (PRC-corrected) by day 28	No serious limitations involved^1^	No serious inconsistency^2^	Direct^3^	Serious imprecision (−1)^4^	RR: 0.45 (0.20 to 1.01); *P* value = 0.05. This summary result indicates that DP treatment was associated with 55% reduction in risk for actual treatment failure compared with AL; highest plausible reduction is 80%. Upper CI limit includes number of no effect, 1	Moderate quality: (+3)

RCT	2,340 (number of studies = 4)	Total treatment failure (PRC-corrected) by day 42	No serious limitations^5^	Serious inconsistency^6^ (−1) [*I* ^2^ = 70]	Direct^3^	Serious imprecision (−1)^7^	RR: 0.56 (0.27 to 1.14); *P* value = 0.11. Pooled RR indicates that DP treatment was associated with a 44% reduction in risk of treatment failure with highest plausible reduction being 73% compared with AL. Upper CI limit crosses line of no effect and includes 14% risk for treatment failure.	Low quality: (+2)

RCT	2,340 (number of studies = 4)	PCR-new *falciparum* infections by day 28	No serious limitations^8^	No serious inconsistency^9^ [*I* ^2^ = 0.00]	Direct^10^	Precise^11^	RR: 0.21 (0.14 to 0.32); *P* value < 0.001. This pooled result means that when DP is used for treatment of *falciparum* malaria it is able to reduce risk of new infections by 79% relative to AL within 28 days. Plausible risk reduction ranges from 68% to 86% as defined by the 95% CI. Result is statistically significant.	High quality: (+4)

RCT	2,662 (number of studies = 4)	PCR-new *falciparum* infections by day 42	No serious limitations^8^	Serious Inconsistency (−1)^12^ [*I* ^2^ = 83]	Direct^7^	No Serious Imprecision^13^	RR: 0.56 (0.34 to 0.91); *P-*value *=* 0.02. The pooled RR means that treatment of *falciparum* malaria using DP is associated with 44% reduction in risk for new infection within 42 days post treatment compared with AL. Range of reduction is between 9% and 66%. Result is statistically significant.	Moderate quality: (+3)^14^


Note: RR = risk ratio, RCT = randomized control trial, CI = confidence interval.

^1^No serious limitations: randomization and concealment were judged to low risk of bias; laboratory personnel and investigators who assessed study outcome were all blinded to avoid measurement bias.

^2^No serious inconsistency: heterogeneity as indicated by *I* ^2^ was 47 and this is classified as moderate heterogeneity.

^3^Directedness: all trials used in the analysis were conducted in countries in sub-Sahara Africa where malaria falciparum transmission is mostly high.

^4^Precision: there is an imprecision because the 95% CI crosses line of no difference and includes increased risk of 1%.

^5^No serious limitations: randomization and concealment were judged to pose low risk of bias; laboratory personnel and investigators who assessed study outcome were all blinded to avoid measurement bias.

^6^Serious inconsistency: there is a substantial statistical heterogeneity as indicated by a very high value of the *I* ^2^ of 70.

^7^Precision: there is a serious imprecision because the interval of the 95% CI crosses line of no effect and also involves an increased risk of 14% for actual treatment failure within 42 days after treatment.

^8^No serious limitations: randomization and concealment were judged to pose low risk of bias and laboratory personnel and investigators were blinded.

^9^No important inconsistency: there was no statistical heterogeneity as shown in the *I* ^2^ being 0.00.

^10^Directedness: there is no important indirectedness because all trials used in the analysis were conducted in countries in sub-Sahara Africa where malaria falciparum transmission is mostly high.

^11^Precision: there is no imprecision in 95% CI of the pooled RR because the CI did not cross the line of no difference hence all benefits were in favour of DP than AL.

^12^Serious inconsistency: *I* ^2^ = 83 implying that there is a substantial statistical heterogeneity.

^13^Precision: there is no imprecision in 95% CI of the pooled RR because the CI did not cross the line of no difference; hence all benefits were in favour of DP than AL.
